# Inhibition of JAK-STAT ERK/MAPK and Glycogen Synthase Kinase-3 Induces a Change in Gene Expression Profile of Bovine Induced Pluripotent Stem Cells

**DOI:** 10.1155/2016/5127984

**Published:** 2016-01-06

**Authors:** Luis F. Malaver-Ortega, Huseyin Sumer, Jun Liu, Paul J. Verma

**Affiliations:** ^1^CSIRO Health & Biosecurity, East Geelong, VIC 3219, Australia; ^2^Monash Institute for Medical Research, Monash University, Clayton, VIC 3168, Australia; ^3^Faculty of Science, Engineering and Technology, Swinburne University of Technology, Hawthorn, VIC 3122, Australia; ^4^Department of Materials Engineering, Monash Institute of Medical Engineering, Faculty of Engineering, Monash University, Clayton, VIC 3168, Australia; ^5^South Australian Research & Development Institute (SARDI), Turretfield Research Centre, Rosedale, SA 5350, Australia

## Abstract

Pluripotent stem cells (PSCs) fall in two states, one highly undifferentiated, the naïve state, and the primed state, characterized by the inability to contribute to germinal lineage. Several reports have demonstrated that these states can be modified by changes to the cell culture conditions. With the advent of nuclear reprogramming, bovine induced pluripotent stem cells (biPSCs) have been generated. These cells represent examples of a transient-intermediate state of pluripotency with remarkable characteristics and biotechnological potential. Herein, we generated and characterized biPSC. Next, we evaluated different culture conditions for the ability to affect the expression of the set of core pluripotent transcription factors in biPSC. It was found that the use of 6-bromoindirubin-3-oxime and Sc1 inhibitors alone or in combination with 5-AzaC induced significantly higher levels of expression of endogenous* REX1*,* OCT4*,* NANOG*, and* SOX2*. Furthermore, LIF increased the levels of expression of* OCT4* and* REX1*, compared with those cultured with LIF + bFGF. By contrast, bFGF decreased the levels of expression for both* REX1* and* OCT4*. These results demonstrate that the biPSC gene expression profile is malleable by modification of the cell culture conditions well after nuclear reprogramming, and the culture conditions may determine their differentiation potential.

## 1. Introduction 

The pursuit of the derivation of pluripotent stem cells (PSC) from livestock species has been one of the major goals for the agribiotech industry. Although some reports demonstrate that it is possible to maintain primary isolates of Embryonic Stem Cell (ECS) in cattle for extended periods of time, a truly, well-characterized cell line is yet to be obtained [1, 2]. This lack of a “gold standard” cell line for the specific case of cattle is due mainly to a level of uncertainty regarding what markers and culture conditions define and maintain pluripotency for* Bos* genus [3]. More recently, with the advent of the nuclear reprogramming technique developed by Yamanaka et al. in 2006, this approach was modified to produce PSC from bovine adult tissues with the aforementioned advantages over ESC for future applications in livestock [4].

Subsequent reports have demonstrated that it is feasible to obtain putative biPSC [5–9]. These putative biPSCs, although they have not yet been examined for the ability to fulfil all the criteria necessary to be called “bone fide” iPSC, for example, contribution to the germ cell lineage, are examples of a transient-intermediate state of pluripotency with remarkable pluripotency characteristics and biotechnological potential [10]. Furthermore, a partially reprogrammed state, similar to the one described so far in iPSC from bovine species, could be practical to the extent that it only requires lower levels of transgene expression achievable with nonintegrative approaches as has been exemplified in human [11].

One of the principal challenges with generation of iPSCs, especially for species other than mouse and human, is the cues necessary for the culture system to maintain the cells after being in a pluripotent, autorenewal state.

Culture conditions affect the pluripotential of PSC and could even revert cells to a more primitive and undifferentiated state [12]. The JAK-STAT, Wnt, and the extracellular signal-regulated kinase (ERK)/mitogen-activated protein kinase (MAPK) ERK/MAPK pathways, vital to sustain and promote pluripotency and self-renewal, can be targeted pharmacologically* in vitro* in order to increase rate of ESC isolation or nuclear reprogramming [7, 13–15]. Further, inhibition of these pathways by the use two inhibitors (2i) promotes a naïve-like state in human iPSC and ESC, demonstrated by an epigenetic reversion and maintenance of a pre-X inactivation state in female lines [16] and, in the case of the mouse, the reversion of EpiSC to a ESC-like state where cells exhibited chimerism competence [17]. The compound 6-bromoindirubin-3′-oxime (Bio) inhibits glycogen synthase kinase-3 (GSK-3) activity by binding the ATP pocket of the kinase and inhibiting the phosphorylation on Tyr276/216 necessary for GSK3 activation, leading to a reduced *β*-catenin phosphorylation [18]. Bio has been shown to allow mouse cells to maintain an undifferentiated state in a feeder free system [19]. However, its effect is not enough to sustain pluripotency in the absence of LIF [19]. Bio also increases efficiency of ESC established from C57BL/6 (B6) and BALB/c mice [20].

Furthermore, it is also possible to inhibit ERK pharmacologically. Its inhibition sustains pluripotency and self-renewal even in the absence of LIF, feeder layers, and serum [21, 22]. Additionally it mediates conversion from the EpiSCs (primed) to the ESC (naïve) state [16, 23], increases the rate of ESC isolation in rats, an example of a refractory species [24, 25], and supports long-term culture of robust human [26] and mouse [22] ESC.

Finally, 5-azacytidine (5-AzaC) and 5-aza-2′-deoxycytidine (Decitabine) are irreversible inhibitors of DNA methyl transferases (DNMTs) that, at low doses, induce hypomethylation, avoiding cytotoxicity [27, 28]. Previous reports have demonstrated that the use of Decitabine or 5-AzaC during isolation of ESC increases the number of colonies obtained in mouse [29] and is also useful for the isolation of putative bESC [30]. Moreover, it has been used to increase nuclear reprogramming efficiency during iPSC generation in human and mouse [31]. These findings together back up the idea that, manipulating the pathways chemically during isolation of primary cells and reprogramming, it is also possible to support, induce, and promote a more undifferentiated state characterized by high rates of self-renewal and pluripotency.

## 2. Material and Methods

### 2.1. Generation of biPSC

To induce pluripotency in adult bovine fibroblasts, we used ectopic expression of Yamanaka's factors: Oct4, SOX2, KLF4, and c-MYC, in addition to NANOG; the latter is required for stable reprogramming in bovine species [5]. Briefly, GP2-293 cells were cultured in 100 mm dishes until 80% confluence and cotransfected with complexes of 54 *μ*L of FuGENE 6, 9 *μ*g pCMV-VSV-G (Addgene ID: 8454), and 9 *μ*g of either pMXs-hOCT4 (ID 17964), pMXs-hSOX2 (ID 17965), pMXs-hcMYC (ID 17966), pMXs-hKLF4 (ID 17967), and pMXs-hNANOG (ID 18115) or CAG-GFP (control). After 15-minute incubation in DMEM, the complex was added dropwise into the dishes. At 24 hours the medium was changed and fresh medium was added. At 48 hours after transfection the medium containing viral particles was collected and filtered using Millex HV filter units (0.45 *μ*m) and added to bAF 100 mm tissues culture dishes previously prepared with 150,000 cells per plate. The same procedure was repeated at 48 hours. Transfection efficiency (TE), defined as the estimate percentage of cells receiving all reprogramming factors, was estimated using EGFP expression and calculated using the formula: TE = GFP · gfp^*n*−1^, where GFP is the percentage of GFP positive cells (control), f is that percentage expressed as a decimal, and *n* is the number of transcription factors utilized during reprogramming. Cells were observed for four weeks. Colonies were passaged manually on mitotic inactivated murine embryonic fibroblast feeder cells for the first ten passages followed by enzymatic passage with a solution of 4 mg/mL of Dispase (Gibco) subsequently. Reprogramming efficiency (RE) was expressed as a percentage and calculated by the formula: RE = (biPSCs colonies)/(initial cells × TE) × 100.

### 2.2. Culture Conditions

Cell lines at passages 24–26 were cultured under defined conditions corresponding to the eight treatments summarized in Table 1. The biPSC media consisted of Minimum Essential Medium Alpha (MEM-*α*) with L-glutamine ribonucleosides and deoxyribonucleosides, Fetal Calf Serum 20% (JRH Bioproducts), GlutaMAX 2 mM, Nonessential Amino Acids (NEAA) 10 *μ*M (Gibco), Human recombinant LIF 5 ng/mL (Sigma), recombinant human Basic Fibroblast Growth Factor (bFGF) 10 ng/mL (Invitrogen), 2-Mercaptoethanol 55 *μ*M, and Penicillin-Streptomycin (25 units and 25 *μ*g, resp., Invitrogen). The media were supplemented with the corresponding inhibitor at the described concentration, and the biPSC media were supplemented with the corresponding inhibitor at the described concentration (Table 1). In order to evaluate the requirements concerning growth factors, bFGF and LIF, each was removed in different treatments. Fresh media were prepared and changed twice a week; biPSC colonies were treated during six passages and evaluated at passages two, four, and six.

### 2.3. biPSC Characterization

For immunostaining, cells were fixed with 100% ice-cold ethanol for 10 min and then stained as described previously [5]. Primary antibodies used were anti-SSEA-1 (MC480, Millipore), anti-SSEA-3 (MAB4303, Millipore), anti-SSEA-4 (MC-813-70, Millipore), anti-Tra-1-60 (MBA4360, Millipore), anti-Tra-1-60 (MBA4381, Millipore), anti-Oct4 (N-19, Santa Cruz), anti-Oct4 (C-10, Santa Cruz), and anti-Nanog (Abcam, ab80892). Secondary antibodies used were 5 goat anti-mouse IgM Alexa-Fluor-488, goat anti-rabbit IgG Alexa-Fluor-594, and goat anti-mouse IgG Alexa-Fluor-594. Alkaline phosphatase assay was determined using Alkaline Phosphatase Detection Kit (Millipore, SCR004) following manufactures' instructions. For* in vitro* differentiation, cells were detached by enzymatic digestion and 1 × 10^6^ cells/well were plated in low attachment six-well plates for 15 days with differentiation media [32]. The differentiation media consisted of *α*-MEM supplemented with foetal bovine serum (JHR 20%), GlutaMAX (Invitrogen 1 : 100), Nonessential Amino Acids (Gibco 1 : 100), Penicillin-Streptomycin (Sigma 1 : 200), Mercaptoethanol (Gibco 1 : 1000), and ITS (Gibco 1 : 100). Teratoma formation was evaluated 8 weeks after IM injection of 5 × 10^6^ cells into the thigh muscle of the hind legs of 8–10-week-old severe combined immune-deficient (SCID) mice.

### 2.4. RT-PCR, Quantitative (qPCR), and Data Analysis

Standard RT-PCR reactions were performed using GoTaq Green Master Mix. qPCR reactions were performed using Power SYBR Green PCR Master Mix (Invitrogen). Analyses were performed in duplicate (technical replicate). mRNA relative fold change values were calculated as the X ± SD  ΔΔct values after normalization against the calibrator [33] (endogenous gene* GAPDH*). When comparison between treatments was pertinent, data was analysed using a two-way ANOVA, Mann-Whitney *U* test, or Student's *t*-test. Differences were considered statistically significant where *p* < 0.05.

## 3. Results and Discussion

### 3.1. Generation and Characterization of biPSC

**Figure 1:**
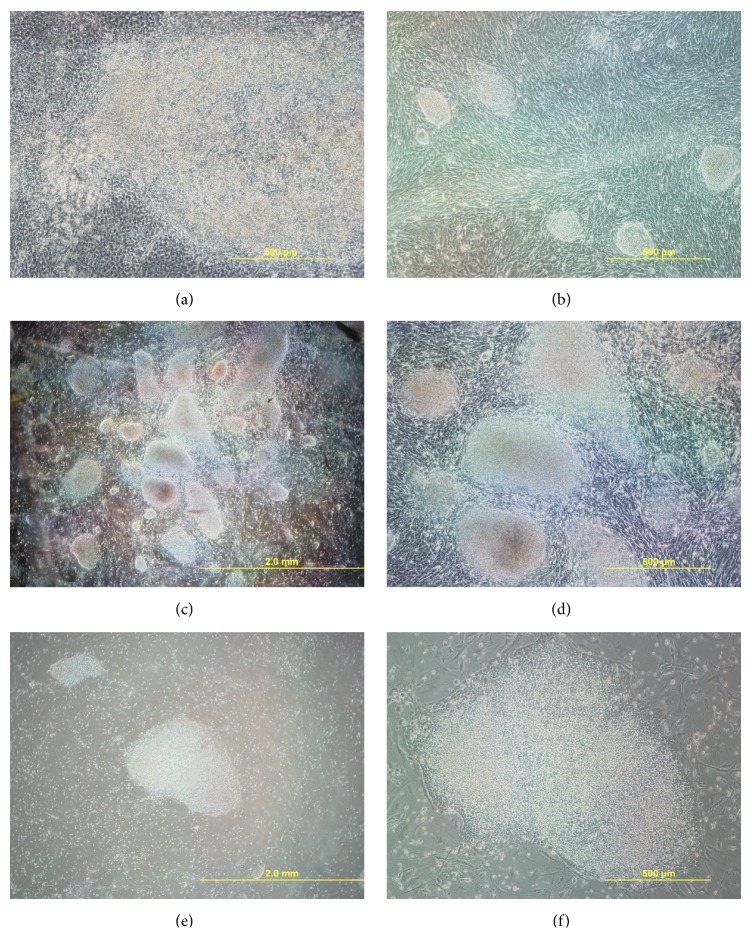
Isolation of biPSC colonies. (a) Initial morphology changes in the original plate were noticeable after two weeks of culture. (b) Well-defined colonies growing on the fibroblast-like background. Large colonies showing a dome shaped (c-d) or flat morphology (e-f).

After 21 days in culture colonies appeared (Figures 1(a) and 1(b)); however, noticeable changes in the morphology were detected as early as two weeks after the last round of infection. The colonies showed distinctive colony morphology compared with fibroblasts visible in the background. TE was 68.2%  ±  9.2 on average, with a reprogramming efficiency of 1.73%  × 10^−4^  ± 1.33 × 10^−5^, considerably lower in comparison with RE from 10 to 100% obtained in mouse by additional modifications to the reprogramming protocol [34, 35].

All noticeable colonies observed were expanded. Morphologically, all colonies obtained fell in one of two categories, based on their morphology, both easily identifiable from the fibroblast-like background. The first is described as large colonies dome shaped (Figures 1(c) and 1(d)). These colonies were initially shiny, but the brightness decreased as they increased in diameter. This morphology resembled that of murine ESC.

The second category appears more close to what has been described for human ESC and murine EpiSC. They were flat well-spread colonies with regular edges and without signs of differentiation. Boundaries between cells were not easily identifiable (Figures 1(e) and 1(f)). Furthermore, although each colony was separately expanded from the original plate, during subsequent passages both morphologies appear to arise from the same clone, showing a degree of variability between passages.

From the colonies isolated, one clone was selected randomly, from each individual line, for further characterization, for a total count of three cell lines.

**Figure 2:**
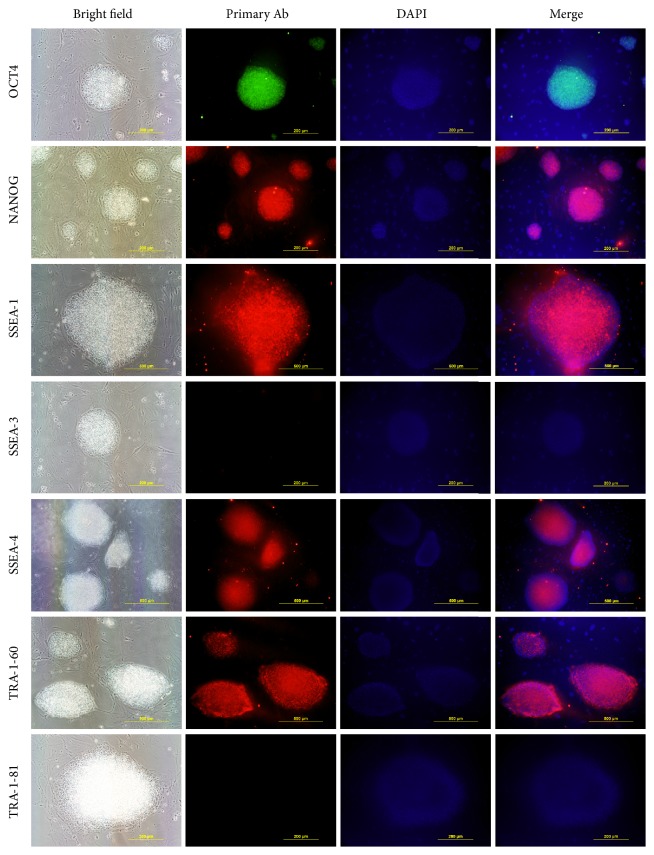
Pluripotency markers immunostaining at early passage. Cells at passage 12 were positive for the nuclear markers OCT4 and NANOG and the surface markers SSEA-1, SSEA-4, and TRA-1-60. None of the colonies were positive for SSEA-3 or TRA-1-81.

First, the expression of the pluripotent markers OCT4 and NANOG was confirmed by immunofluorescence analysis of all colonies. The colonies were also positives for SSEA-1 and SSEA-4 along with TRA-1-60. The expressions of SSEA-3 and TRA-1-81 were also evaluated and found to be absent (Figure 2) and the same profile of expression was maintained over 44 passages. All cell lines were weakly alkaline phosphatase positive and euploid male, displaying a normal 60 XY karyotype without gross chromosomal abnormality in terms of number or G banding staining (Figure S1 in Supplementary Material available online at http://dx.doi.org/10.1155/2016/5127984). When we evaluated the expression of the classic surface markers used for ESC characterization by immunostaining, the cell lines were positive for NANOG, OCT4, SSEA-1, SSEA-4, and TRA-1-60 but negative for SSEA-3 and TRA-1-81. The surface markers SSEA-1 and SSEA-4 have been widely associated with isolation of putative ESC in bovine [1]. The expressions of NANOG and OCT4 are less reliable based on immunostaining alone, as the translated protein could have come from exogenous gene expression. Comparing our results with other works on bovine nuclear reprogramming, only SSEA-1, OCT4, and NANOG appear consistent as makers for biPSC [7, 8].

**Figure 3:**
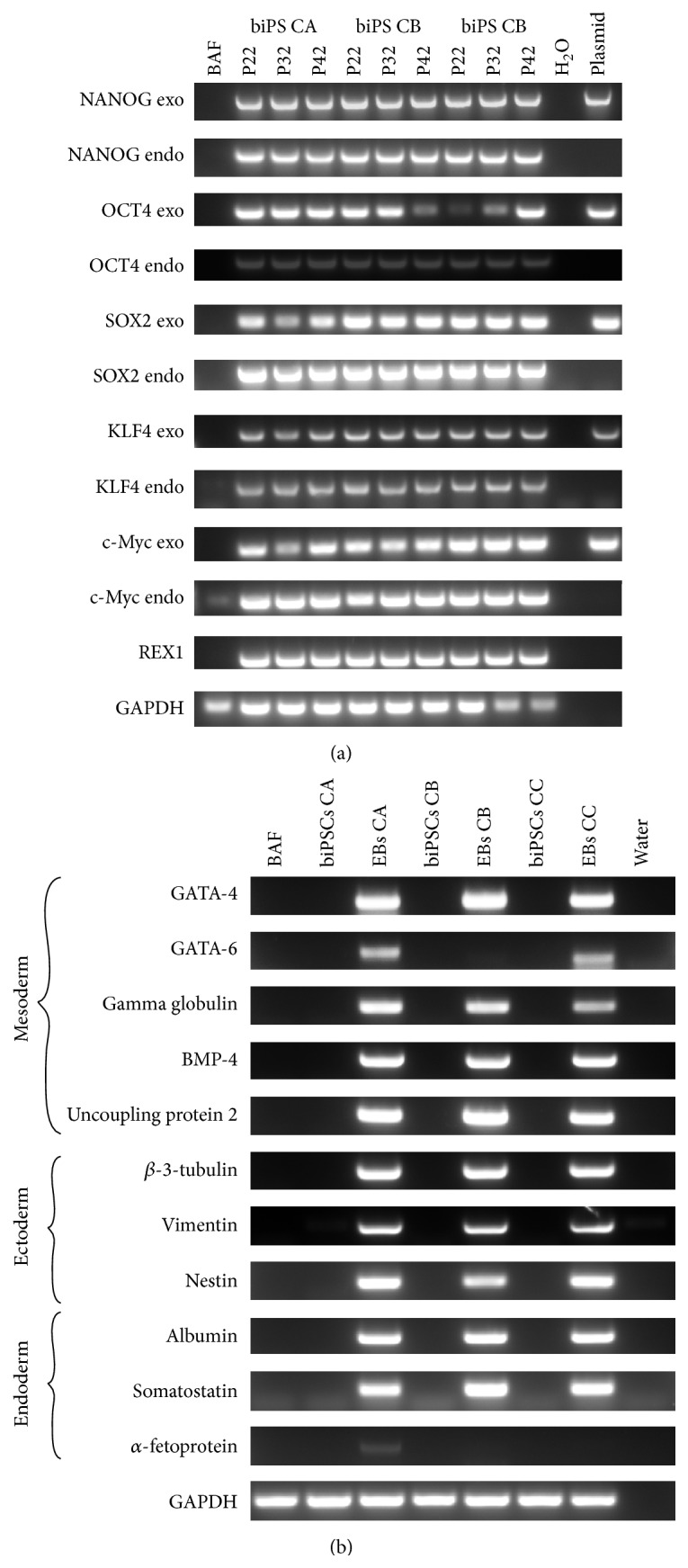
Endogenous and exogenous gene expression before and after differentiation. (a) Gene expression by RT-PCR of the exogenous (exo) and endogenous (endo) genes ONSKcM used during reprogramming and REX-1. Plasmids are used as control. (b) Cells were differentiated by EBs and a set of different genes representative of all three germ cell layers were evaluated by RT-PCR.

The endogenous and exogenous expression of the core set of reprogramming factors* OCT4*,* SOX2*,* KLF4*,* C-Myc*,* NANOG*, and* REX1* were evaluated by RT-PCR. Cells expressed reprogramming genes both ectopically and endogenously. With the exception of endogenous* NANOG* at early passages (data not showed), all cell lines evaluated expressed endogenous* OCT4*,* SOX2*,* KLF4*,* C-Myc*,* NANOG*, and* REX1* (Figure 3(a)). After reprogramming, the cells expressed constantly the ectopic factors:* OCT4*,* SOX2*,* KLF4* and* C-Myc*, and* NANOG*; as seen in previous reports on cattle iPSC, they did not silence the exogenous genes. This poses the question on what extent the cells depend on the exogenous and not the endogenous gene to maintain pluripotency, a pivotal requirement of truly reprogrammed cells. Interestingly, cells also expressed* REX1*, a pluripotent cell marker not included in the reprogramming cocktail.

Differentiating embryoid bodies from the three cell lines was evaluated for the expression of the genes* GATA4* and* GATA6*,* gamma globulin*,* BMP4*,* uncoupling protein 2 tubulin*,* beta 3*, and the intermediate filaments:* vimentin*,* nestin*,* somatostatin*, and* albumin* and its foetal equivalent *α-fetoprotein*. The expression of these genes was not detectable in the parental cell line and biPSC before differentiation (Figure 3(b)).

**Figure 4:**
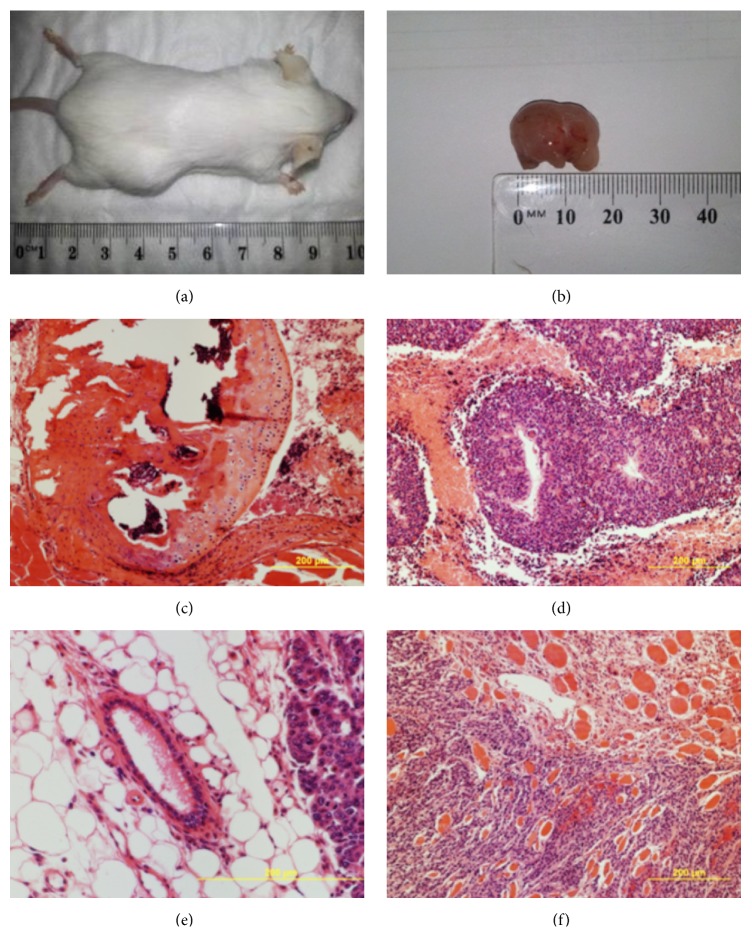
biPSC differentiation* in vivo*. Teratomata were induced in female mice (a-b) and they were dissected and stained (H&E). Histological analysis showed cartilage (c), neural rosettes (d), adipose (e), and muscle (f) differentiation.

Finally, the* in vivo* potential to generate teratomata was evaluated in the three lines. Six-week postinoculation outgrowths around 1 cm diameter were evident (Figures 4(a) and 4(b)). All three cell lines produce robust teratomas with histological analysis demonstrating cell differentiation to cell types including cartilage, neural rosettes, adipose tissue, and muscle cells, indicative of the three germ layers (Figures 4(c)–4(f)). This is a key demonstration of the robust pluripotency displayed for the cell lines generated.

### 3.2. Effect of Inhibitors in Endogenous and Exogenous Expression of Reprogramming Factors

Next, we evaluated different culture conditions able to sustain the endogenous expression of factors related to pluripotency. Previously it has been described that the inhibition of the different pathways increases the rate of isolation of ESC in mouse and human. In this vein, two different cell lines at passages 25 and 28 (*n* = 4), respectively, were cultured for up to six passages after characterization under the conditions described in Table 1. The expression of endogenous and exogenous genes was evaluated by qRT-PCR over the six passages.

**Figure 5:**
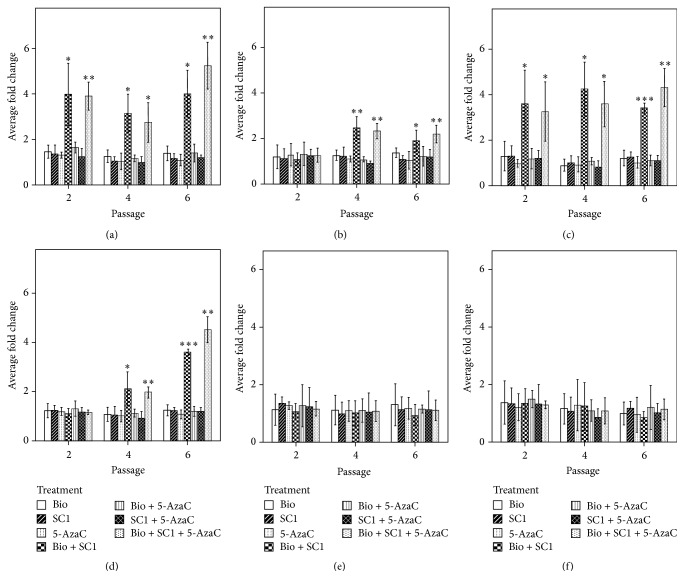
Effect of inhibitors on the expression endogenous genes. REX (a), NANOG (b), OCT4 (c), SOX2 (d), c-Myc (e), and KLF4 (f) genes. Results expressed as the mean of fold change ±2 standard deviation (SD) relative to control (no inhibition). ^*∗*^ *p* < 0.05, ^*∗∗*^ *p* < 0.01, and ^*∗∗∗*^ *p* < 0.001.

There were no apparent changes in terms of gross morphology between treatments. However, the expression of the ESC markers was upregulated with two inhibitors 2i and 2i combined with DNMTs inhibition (Di). In the case of* REX 1* this effect appears as early as after two passages. Although there was no statistically significant difference between 2i and 2i + Di (*p* ≥ 0.05), 2i + Di showed a significant increase against control (5.2 ± 1.0-fold; *p* < 0.01) compared with 2i alone (4.0 ± 1.0-fold; *p* < 0.05) after six passages (Figure 5).

*NANOG*,* OCT4*, and* SOX2*, members of the core pluripotent transcription factors, showed significant changes in endogenous expression under 2i and 2i + Di treatment. In the case of* NANOG* this effect was rather discrete but significant for 2i (2.47 ± 0.4-fold; *p* < 0.01) (1.9 ± 0.4-fold; *p* < 0.01) and 2i + Di (2.3 ± 0.3-fold; *p* < 0.05) (2.1 ± 0.3-fold; *p* < 0.01) after four and six passages, respectively.

Among the factors used for reprogramming,* OCT4* showed the earliest changes in level of expression with an increase, after two passages, of (3.5 ± 1.4-fold; *p* < 0.05) and (3.2 ± 1.2-fold; *p* < 0.05) using 2i and 2i + Di, respectively. Finally,* SOX2* exhibited the highest upregulation after six passages, once again under 2i and 2i + Di only with average fold changes of (3.5 ± 0.1-fold; *p* < 0.001) and (4.4 ± 0.5-fold; *p* < 0.01) correspondingly (Figure 5). In contrast to what is seen for the endogenous expression, ectopic expression levels were not affected and they were constant during six passages (data not showed).

### 3.3. Analysis on biPSc Dependency on LIF or bFGF Signalling

A common practice during the isolation and generation of PSC from species different to human and mouse is the combined use of two growth factors, bFGB and LIF, in order to provide both signals required to maintain the naïve and primed states represented by mouse and human ESC, respectively [10]. The idea behind this is to provide the required growth factors, for both stages, based on the premise that these two stages are less clearly defined in species different to rodents and humans. Hence, we decided to analyse the expression of the transcriptional core of pluripotency* OCT4*,* NANOG*,* SOX2*, and* REX1* combined with morphology changes of the biPSC generated under either LIF or primed bFGF culture conditions.

Cells were maintained for up to eight passages under either LIF or bFGF stimulus. The combination of both LIF and bFGF was used as a control. After eight passages cells did not show any apparent changes in gross morphology with any differences observed when compared with the control (data no showed).

**Figure 6:**
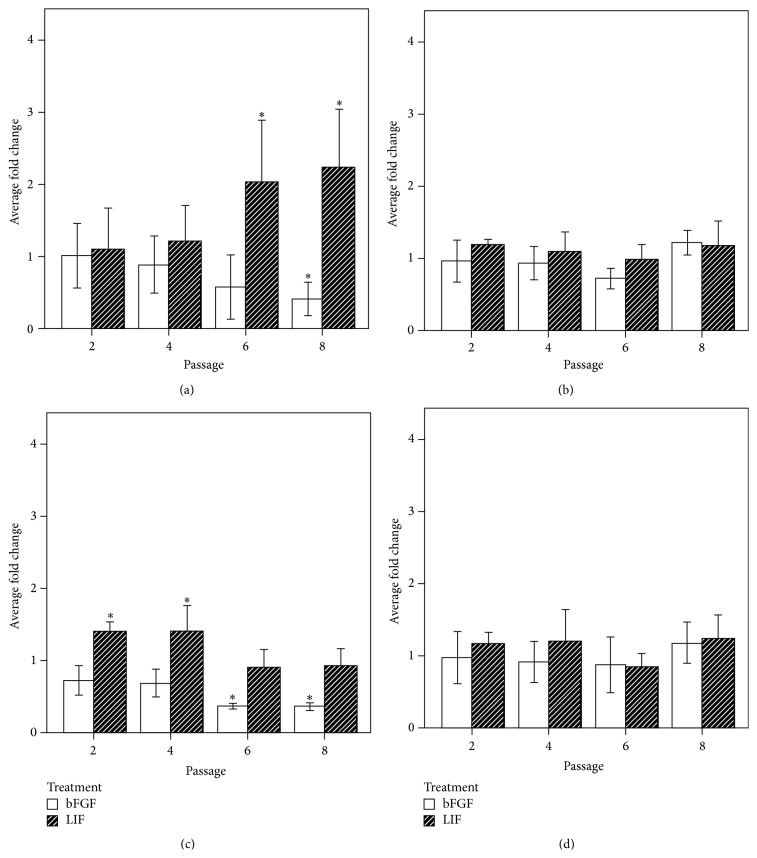
Effect of LIF and bFGF on endogenous genes: REX-1 (a), NANOG (b), OCT4 (c), and SOX2 (d) genes. Results expressed as the mean of fold change ±2 standard deviation (SD) relative to control (LIF and bFGF). ^*∗*^ *p* < 0.05.

However, when the levels of RNA expression of the endogenous and exogenous* OCT4*,* NANOG*,* SOX2*, and* REX1* were determined, differences in the profile of gene expression were observed.* REX1*, under bFGF stimulus, showed a steady and gradual decrease with the levels of expression significantly reduced after eight passages (0.4 ± 0.1-fold; *p* < 0.05). Conversely, under LIF alone stimulus the level of expression increased up to two times in the same period of time (2.2 ± 0.5-fold; *p* < 0.05) (Figure 6(a)).

The second transcription factor affected by the use of different growth factors during culture was endogenous* OCT4*. Similar to the response of* REX1* under bFGF stimulus, the level of* OCT4* decreased in presence of bFGF after six (0.3 ± 0.04-fold; *p* < 0.05) and eight (0.3 ± 0.05-fold; *p* < 0.05) passages, an effect not related to the number of passages (Figure 6(c)).

The effect of LIF on* OCT4* expression was less distinct as the analysis showed a significant increase during the first four passages (1.4 ± 0.13-fold; second passage, and 1.4 ± 0.35-fold; fourth passage; *p* < 0.05) which dropped from passage five back to control levels (Figure 6(c)). There was no effect on the expression profile of the exogenous* NANOG*,* OCT4*, and* SOX2* or endogenous* NANOG* or* SOX2* across different cell culture passages (Figures 6(b) and 6(d)).

## 4. Conclusions

We demonstrated that, even after induced cell reprogramming, the gene expression profile is malleable by modification of the cell culture conditions. Using simultaneous inhibition of GSK-3, ERK1, and Raps-GAP it is possible to increase the expression of at least four genes of the core transcription complex responsible for pluripotency. Levels of* REX1*,* NANOG*,* OCT4*, and* SOX2* were increased after inhibition of the mentioned pathways.* REX1* showed the most dramatic effect with an increase of nearly five times the levels in controls.* NANOG*,* OCT4*, and* SOX2* also showed significant upregulation, although the increases were modest compared with* REX1* (twice that of control). This upregulation effect was limited to the endogenous genes, which suggests that the effect was exerted at the transcriptional control level taking into account that a viral promoter mediates ectopic expression.

Using similar inhibitors it has been widely reported that both reprogramming and ESC isolation are augmented with the highest RE and isolation success, respectively [13, 22, 24]. These results support our hypothesis that intervention in culture conditions could affect reprogramming status in bovine as it was demonstrated before for mice where established cell lines could change their expression profile to a more “pluripotent state” following GSK-3, ERK1, and Ras-GAP inhibition [23]. In our work, DNMTs inhibition had no effect either alone or in combination with inhibitors, on the expression of the evaluated genes.

Finally, we evaluated the use of LIF and bFGF on long-term culture and gene expression of biPSC. These two factors are believed to sustain one or the other of the two characteristic states: naïve and primed, respectively. Under LIF stimulus alone the levels of* REX1* and* OCT4* increased, and by contrast their levels reduced under bFGF culture. Taking into account that both relative measurements are based on the expression under normal culture condition (with a combination of LIF and bFGF) these findings suggest that biPSCs are responsive to those stimuli well after induced reprogramming and the culture conditions may determine the eventual gene expression profile on the biPSC and consequently their pluripotency and differentiation potential.

## Supplementary Material

Figure S1: Alkaline phosphate activity in the biPSC colonies produced using alkaline phosphatase activity was determined using the Alkaline Phosphatase Detection Kit (Millipore, SCR004) following manufactures' instructions. The colonies were weakly positive. All the cell lines displayed normal karyotypes corresponding to male individuals.
Table S1: The list of primers used during the study is presented. The primers were selected to distinguish between endogenous and ectopic gene expression of the genes evaluated.

## Figures and Tables

**Table 1:** Summary of the different treatments used to evaluate the effect of pathway inhibition on gene expression profiles. 5-Azacytidine (5-AzaC), glycogen synthase kinase-3 (GSK-3), extracellular signal-regulated kinase (ERK)/mitogen-activated protein kinase (MAPK), and DNA methyltransferases (DNMTs)^*∗∗∗*^.

	GSK3	ERK/MAPK	DNMT (Di)	GSK3 + ERK/MAPK (2i)	GSK3 + DNMT	ERK/MAPK + DNMT	2i + Di	Control
Bio	1 *µ*M	*∗∗∗*	*∗∗∗*	1 *µ*M	1 *µ*M	*∗∗∗*	1 *µ*M	*∗∗∗*
SC1	*∗∗∗*	0.5 *µ*M	*∗∗∗*	0.5 *µ*M	*∗∗∗*	0.5 *µ*M	0.5 *µ*M	*∗∗∗*
5-AzaC	*∗∗∗*	*∗∗∗*	5 *µ*M	*∗∗∗*	5 *µ*M	5 *µ*M	0.5 *µ*M	*∗∗∗*
