# Supplementary material for: Inhibition of JAK-STAT ERK/MAPK and Glycogen Synthase Kinase-3 Induces a Change in Gene Expression Profile of Bovine Induced Pluripotent Stem Cells
Source: Stem Cells Int. 2016 Jan 6;2016:5127984. doi: 10.1155/2016/5127984 (PMC4736581; doi:10.1155/2016/5127984)
Supplement: Supplementary file 1 — Figure S1: Alkaline phosphate activity in the biPSC colonies produced using alkaline phosphatase activity was determined using the Alkaline Phosphatase Detection Kit (Millipore, SCR004) following manufactures' instructions. The colonies were weakly positive. All the cell lines displayed normal karyotypes corresponding to male individuals. Table S1: The list of primers used during the study is presented. The primers were selected to distinguish between endogenous and ectopic gene expression of the genes evaluated. [file 5127984.f1.pdf]

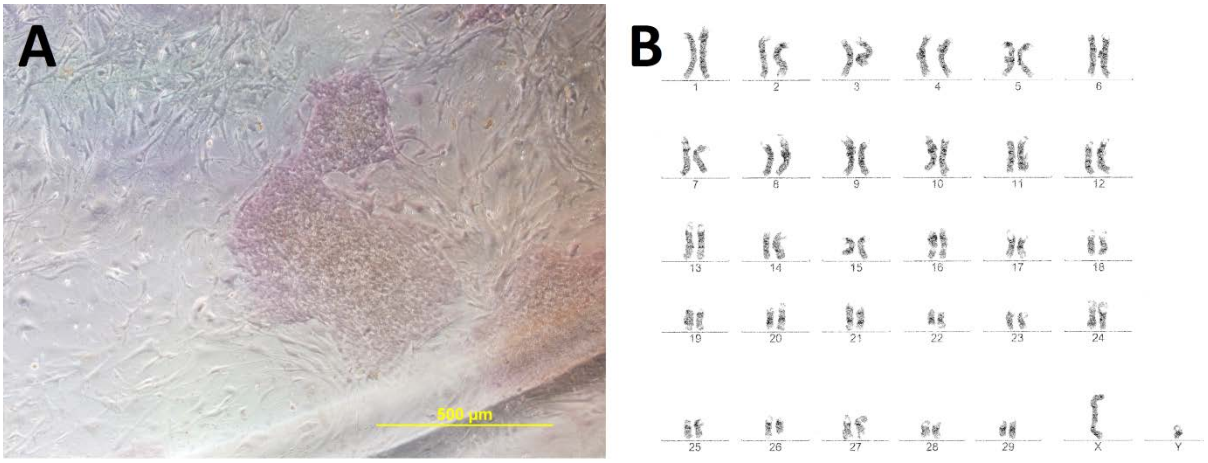

Figure S1 (A) Alkaline phosphate assay on biPSC colonies, Cells were weakly positive. (B) Normal bovine karyotype displayed by the biPSC generated.

Table S1 List of primers

Primer	Sequence	Length	Tm	GC%	P.S	t°	Ref
pMxhOCT4	*CTAGTTAATTAAGAATCCCAGTG*	23	46.6	34.8	300.0	58	Sumer et al. 2011
	CACTAGCCCCACTCCAACCT	20	55.2	60.0			
pMxhSOX2	*CTAGTTAATTAAGGATCCCAGG*	22	53.1	40.9	300.0	58	Sumer et al. 2011
	TGTTGTGCATCTTGGGGTTCT	21	60.1	47.6			
pMx hKLF4	*ACAAAGAGTTCCCATCTCAAGGTG*	24	60.8	45.8	250.0	58	Sumer et al. 2011
	TCCAAGCTAGCTTGCCAAACCTACAGG	27	66.8	51.9			
pMxhc-MYC	*CTAGTTAATTAAGGATCCCAGTG*	23	54.0	39.1	500.0	58	Sumer et al. 2011
	CAGCAGCTCGAATTTCTTCC	20	57.2	50.0			
pMX-hNanog	*CTAGTTAATTAAGAATCCCAGTG*	23	52.4	34.8	550.0	58	Sumer et al. 2011
	GGGTAGGTAGGTGCTGAGGC	20	61.7	65.0			
SOX2	*CATCCACAGCAAATGACAGC*	20	57.7	50.0	251.0	58	Sumer et al. 2011
	TTTCTGCAAAGCTCCTACCG	20	58.2	50.0			
POU5F1	*GTTCTCTTTGGAAAGGTGTTC*	21	49.3	42.9	313.0	58	Sumer et al. 2011
	ACACTCGGACCACGTCTTTC	20	54.2	55.0			
NANOG	*GTGTTTGGTGAACTCTCCTG*	20	50.6	50.0	308	58	Sumer et al. 2011
	GGGAATTGAAATACTTGACAG	21	46.8	38.1			
KLF4	*GCCCCTAGAGGCCCACTT*	18	54.7	66.7	455	58	Sumer et al. 2011
	CACAACCATCCCAGTCACAG	20	52.7	55.0			
c-MYC	*CGCGGTCGCCTCCTTCTCGCCCAGG*	25	68.7	76.0	412	58	Sumer et al. 2011
	GTCCGGGGAAGCGCAGGGC	19	62.6	79.0			
GAPDH	*ACCCAGAAGACTGTGGATGG*	20	59.0	55.0	247	60	Gad et al, 2012
	ACGCCTGCTTCACCACCTT	19	62.1	57.9			
POU5F1	*GTTTTGAGGCTTTGCAGCTC*	20	58.22	50	182	55	Gad et al, 2012
	CTCCAGGTTGCCTCTCACTC	20	59.75	60			
0ct4 3'UTR	*GGGTTTGTACTAGGGCTTTGGG*	22	60.88	54.55	124	55	Han et al, 2011
	GCATCATTGAACTTCACCTTCCCT	24	61.35	45.83			
Sox2-3'UTR	*GCACGGCCATTAACGGCACAC*	21	65.25	61.9	126	60	Han et al, 2011
	CTCCATGCTGTTTCTTGCTGTCCTC	25	64.04	52			
Nanog 3'UTR	*TCTGTGTCAGTTTGAGGGACAGG*	23	62.07	52.17	234	60	Han et al, 2011
	AACAAGTAAAGCCTCCCTATCCCA	24	61.32	45.83			
Rex1	*CACTGTCCTTCGATTACAACCCCAG*	25	63.31	52	231	60	Han et al, 2011
	CCACGTACTTACTGCTGGAGATGGG	25	64.62	56			
